# Ternary dual *Z*-scheme graphitic carbon nitride/ultrathin metal–organic framework nanosheet/Ag_3_PO_4_ photocatalysts for boosted photocatalytic performance under visible light†

**DOI:** 10.1039/c9ra08292a

**Published:** 2019-12-02

**Authors:** Tomoharu Kusutaki, Hideyuki Katsumata, Ikki Tateishi, Mai Furukawa, Satoshi Kaneco

**Affiliations:** Department of Chemistry for Materials, Graduate School of Engineering, Mie University Tsu Mie 514-8507 Japan hidek@chem.mie-u.ac.jp +81-59231-9425 +81-59231-9425; Mie Global Environment Center for Education & Research, Mie University Tsu Mie 514-8507 Japan

## Abstract

Ternary graphitic carbon nitride/ultrathin metal–organic framework nanosheet/Ag_3_PO_4_ (CNUA) composite photocatalysts were prepared under ultrasonic irradiation in tetrahydrofuran. The aim was to use them as photocatalysts for the degradation of organic pollutants in water. The crystal structure, surface morphology, optical properties, and chemical composition of the photocatalytic materials were investigated using X-ray diffraction, scanning electron microscopy, UV-vis diffuse reflectance spectroscopy, and X-ray photoelectron spectroscopy (XPS). The XPS analysis revealed the formation of Ag nanoparticles, which play an important role as an electronic mediator and photosensitizer in the composite during the synthesis. The photocatalytic activity of the composites in the degradation of 2-chlorophenol (2-CP) under visible light (>420 nm) was evaluated. Among the synthesized photocatalysts, the optimized CNUA with 10 wt% of g-C_3_N_4_ with respect to Ag_3_PO_4_ (CN10UA), exhibited the best photocatalytic performance in the degradation of 2-CP, which was almost decomposed completely upon ∼5 min of visible-light irradiation. Furthermore, the stability of the CN10UA photocatalyst could be maintained at a high level even after four cycling experiments, while that of pure Ag_3_PO_4_ declined significantly. The enhanced photocatalytic performance results from efficient charge separation through the dual *Z*-scheme mechanism involving Ag(0) bridges in the g-C_3_N_4_/Ag/Ag_3_PO_4_ and Ag_3_PO_4_/Ag/UMOFN pathways. The analysis of the photoluminescence of the catalysts also provided evidence for charge transport *via* the dual *Z*-scheme mechanism. In addition, radical scavenging tests confirmed that h^+^ and O_2_˙^−^ are the main radical reactive species responsible for the photodegradation of 2-CP. The findings of this study enhance our understanding of the construction and mechanism of dual *Z*-scheme-type photocatalysts.

## Abstract 2

The enhanced photocatalytic activity of CN10UA results from fast charge transport through dual *Z*-scheme channels.

## Graphical abstract

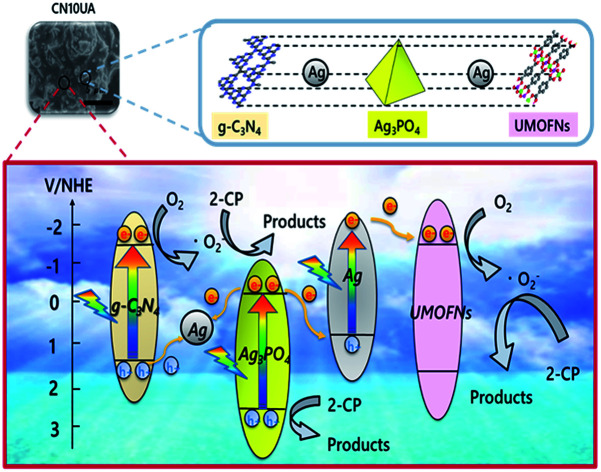

## Introduction

Global issues, such as energy shortage and environmental pollution, are important problems that need to be solved for the sustainable development of modern society. In recent years, photodegradation of organic pollutants in photocatalytic reaction systems has attracted considerable interest as one of the most promising methods for environmental remediation.^1^ Recently, metal–organic frameworks (MOFs), in which metal cations and polydentate ligands are cross-linked, have attracted much interest as promising porous materials,^2–4^ because of their outstanding properties such as a large surface area, high porosity, excellent adsorption selectivity, and chemical stability. In addition, MOFs have also been widely utilized in photocatalysis, separation, gas storage, chemical sensors, and biomedicine; because their properties can be easily modulated by changing the size and chemical environment of the pore space.^5–17^ In particular, studies on the application of MOFs in photocatalysis have received much attention from the scientific community. According to Xamena *et al.*, MOF-5 composed of Zn_4_O_13_ quantum dots and terephthalate linkers, shows excellent photocatalytic activity in the degradation of phenolic molecules under UV light.^18^ Further, Hardi *et al.* reported that MIL-125, constructed from titanium-oxo-hydroxo clusters and dicarboxylate linkers, shows high thermal stability and excellent photocatalytic activity owing to the formation of a Ti(iii)–Ti(iv) mixed valence state in MIL-125 under UV-visible irradiation.^19^ In addition, ultrathin MOF nanosheets (UMOFNs), which are constructed from Ni^2+^, Co^2+^, and terephthalate linkers, exhibit high electrical conductivity, fast charge migration and high percentage of exposed active sites, and can serve as two-dimensional photocatalysts.^20^ Moreover, these properties of bimetal-UMOFNs are superior to those of single-metal-UMOFNs because of the interaction between Ni^2+^ and Co^2+^*via* the bridging O^2−^ and the optimization of the orbitals occupancy in the coordinatively unsaturated metals.^21^ However, the application of these MOFs as photocatalysts is hindered due to some drawbacks in terms of low effective separation of photogenerated charge carriers, poor photostability, and their absorption lying in the UV range and not in the visible one. Therefore, it is important to form a heterojunction between MOFs and visible-light-responsive photocatalysts.^22^

In recent years, a considerable number of studies have been carried out in the field of photocatalysis on graphitic carbon nitride (g-C_3_N_4_), which is a metal-free photocatalyst. It is generally known that g-C_3_N_4_ has excellent properties, such as superior photoelectronic characteristics^23,24^ and high stability in water systems,^25,26^ as well as photocatalytic performance under visible-light irradiation.^27,28^ However, it has insufficient photocatalytic activity for practical application owing to the fast recombination rate of photogenerated electron–hole pairs. Therefore, development of composite photocatalysts based on g-C_3_N_4_is recommended for achieving efficient charge separation.^29^ For example, TiO_2_/g-C_3_N_4_,^30^ WO_3_/g-C_3_N_4_,^31^ ZnO/g-C_3_N_4_,^32^ SnO_2_/g-C_3_N_4_,^33^ graphene oxide (GO)/g-C_3_N_4_,^34^ CdS/g-C_3_N_4_,^35^ AgBr/g-C_3_N_4_,^36^ UMOFN/g-C_3_N_4_,^37^ ZIF-5/g-C_3_N_4_,^38^ and UiO-66/g-C_3_N_4_ (ref. 39) have been reported. These composite photocatalysts show remarkably enhanced photocatalytic performances as compared to that of pure g-C_3_N_4_. This is because the constructed heterojunction system facilitates efficient separation of photogenerated carriers, which could lead to higher photocatalytic performance.

Over the past few years, many researchers have shown interest in Ag_3_PO_4_as a photocatalyst because of its excellent photocatalytic activity under visible-light irradiation. In addition, the photocatalytic performance of Ag_3_PO_4_ can be further improved by modifying its surface properties.^40–43^ There is, however, a particular disadvantage in using Ag_3_PO_4_ as a photocatalyst; an excessive amount of Ag metal is deposited on the surface of Ag_3_PO_4_ due to its photo-corrosion during the photocatalytic reaction. The deposited metallic Ag interferes with charge transfer, light adsorption, and adsorption of organic pollutants. Therefore, the structure and photocatalytic activity of Ag_3_PO_4_ gradually deteriorate. In order to solve this problem, considerable attention has been paid to the development of Ag_3_PO_4_ composite photocatalysts such as g-C_3_N_4_/Ag_3_PO_4_,^44^ ZnO/Ag_3_PO_4_,^45^ MoSe_2_/Ag_3_PO_4_,^46^ TiO_2_/Ag_3_PO_4_,^47^ GO/Ag_3_PO_4_,^48^ HKUST-1/Ag_3_PO_4_,^49^ NH_2_-MIL-125/Ag_3_PO_4_,^50^ and AgBr/Ag_3_PO_4_.^51^ In comparison with pure Ag_3_PO_4_, these photocatalysts show superior photocatalytic performance owing to the formation of heterojunctions.

Furthermore, in recent years, various ternary semiconductors have been developed in order to further improve the photocatalytic performance of binary photocatalysts. The ternary photocatalysts with suitable matching energy band structures provide more efficient charge separation than binary photocatalysts. Wang *et al.* prepared a ternary GO/Ag_3_PO_4_/AgBr composite *via* an *in situ* anion-exchange method, and it showed higher photocatalytic degradation of rhodamine B (RhB) under visible-light irradiation in comparison with those of pure Ag_3_PO_4_ and Ag_3_PO_4_/AgBr.^52^ Yan *et al.* demonstrated that ternary Ag_3_PO_4_/GO/g-C_3_N_4_ photocatalysts synthesized by a chemical precipitation method has superior photocatalytic activity in RhB degradation than single or binary photocatalysts. In this ternary system, Ag_3_PO_4_, GO, and g-C_3_N_4_ serve as the photosensitizer, cocatalyst, and visible-light-driven photocatalyst, respectively, and together they enhance the photocatalytic activity under visible-light irradiation.^53^ Xu *et al.* synthesized a magnetic Ag_3_PO_4_/TiO_2_/Fe_3_O_4_ nanocomposite that presented excellent properties including cycling stability, long-term durability, and effective charge separation because of the heterojunction structure in the ternary system.^54^ As reported by Liu *et al.*, the ternary g-C_3_N_4_/Ag_3_PO_4_/Ag_2_MoO_4_ photocatalysts exhibited efficient charge separation and superior water oxidation under visible-light irradiation owing to the generation of dual *Z*-scheme-type Ag bridges.^55^ According to our previous studies, tetrahedral UMOFN/Ag_3_PO_4_ core–shell photocatalysts have great potential for application in environmental purification technologies.^56^ The synergistic effects of the tetrahedral structure and heterojunction can be harnessed in the formation of useful ternary photocatalysts when combined with a third material. Therefore, the formation of a ternary heterojunction, such as in the g-C_3_N_4_/UMOFNs/Ag_3_PO_4_ system, could lead to further improvement in the photocatalytic activity. However, to the best of our knowledge, there are no reports on ternary g-C_3_N_4_/UMOFNs/Ag_3_PO_4_ composite photocatalysts.

In this study, we developed a new ternary g-C_3_N_4_/UMOFNs/Ag_3_PO_4_ composite photocatalyst to further enhance the photocatalytic activity. The ternary g-C_3_N_4_/UMOFNs/Ag_3_PO_4_ photocatalysts were easily fabricated in a tetrahydrofuran (THF) solution under ultrasound irradiation. The photocatalytic performance of the ternary g-C_3_N_4_/UMOFNs/Ag_3_PO_4_ photocatalysts was evaluated by performing the photodegradation of 2-chlorophenol (2-CP) under visible-light irradiation (>420 nm) in their presence. Furthermore, the crystal structure, optical properties, chemical composition, and surface morphology of the photocatalytic samples were characterized, and the degradation mechanism of 2-CP under the catalysis of ternary g-C_3_N_4_/UMOFNs/Ag_3_PO_4_ photocatalysts was carefully studied in detail.

## Experimental

### Materials and methods

#### Synthesis of photocatalysts

All the reagents used in this study were analytically pure and were used without further purification. For the preparation of tetrahedral Ag_3_PO_4_ particles,^57^ AgNO_3_ (12 mmol) was first completely dissolved in ethanol (80 mL) under ultrasonication. Then a solution of H_3_PO_4_ (20 mL) in ethanol (80 mL) was mixed with the AgNO_3_ precursor solution under ultrasonication for 1 h in dark. Finally, the obtained tetrahedral Ag_3_PO_4_ particles were collected by centrifugation, washed four times with ethanol, and dried under vacuum at 30 °C.

UMOFNs were synthesized according to a previously reported procedure.^20^*N*,*N*-Dimethylformamide (DMF, 32 mL), ethanol (2 mL), and distilled water (2 mL) were mixed by sonication for 10 min. Then, terephthalic acid (0.75 mmol) was added to the mixed solution. Next, CoCl_2_·6H_2_O (0.375 mmol) and NiCl_2_·6H_2_O (0.375 mmol) were simultaneously dissolved in the mixed solution. After the complete dissolution of the two salts, triethylamine (0.8 mL) was rapidly added to the mixture, and it was ultrasonicated for 8 h in a sealed glassware. Finally, the obtained product was centrifuged, washed four times with ethanol, and dried under vacuum at 30 °C to recover a pale pink powder of UMOFNs.

g-C_3_N_4_ was synthesized according to a previously reported procedure.^44^ In a typical synthesis, 10 g of urea was placed in an alumina crucible with a cover, and it was calcined in an electric furnace at 550 °C for 5 h at the heating rate of 20 °C min^−1^. After cooling naturally to room temperature, the obtained g-C_3_N_4_ was ground into a powder for further use.

The ternary g-C_3_N_4_/UMOFNs/Ag_3_PO_4_ (CNUA) composite photocatalysts were prepared *via* ultrasound irradiation in a THF solution. First, the prepared UMOFNs (20 mg) and an appropriate amount of g-C_3_N_4_ were uniformly dispersed in tetrahydrofuran (100 mL) through sonication for 30 min. Then, the prepared Ag_3_PO_4_ tetrahedron (400 mg) was added to the mixed solution, and the mixture was sonicated in the dark for 6 h. The so-obtained CNUA was centrifuged, washed four times with ethanol, and dried under vacuum at 30 °C. The weight ratio of UMOFNs to Ag_3_PO_4_ was optimized according to a previously published method.^56^ A series of CNUA samples were prepared by varying the amount of g-C_3_N_4_in the composite, and samples with 5, 10, 20, 30, 50, and 70 wt% of g-C_3_N_4_ were referred to as CN5UA, CN10UA, CN20UA, CN30UA, CN50UA, and CN70UA, respectively. For comparison, binary composites of UMOFN/Ag_3_PO_4_ (UA) without g-C_3_N_4_ and g-C_3_N_4_/Ag_3_PO_4_ (CNA) with 10 wt% g-C_3_N_4_ and no UMOFNs were also prepared according to the same procedure.

#### Photocatalytic degradation of 2-CP

The photocatalytic performance of the as-prepared catalysts was analysed by evaluating the degradation rate of 2-CP under visible-light irradiation (*λ* > 420 nm). First, the photocatalytic material (30 mg) was added to an aqueous solution of 2-CP (30 mg L^−1^) in a Pyrex reactor. Then, the aqueous 2-CP solution including the photocatalytic material was stirred for 30 min to attain adsorption–desorption equilibrium. Next, the reaction system was illuminated with a 300 W Xe lamp (MAX-303, Asahi Spectra) with a short wavelength cut-off filter (L-42, HOYA) as a visible light source. Then, 2 mL aliquots of the reaction mixture in the suspension were retrieved at preset intervals. After centrifugation, the obtained solution was analysed by high-performance liquid chromatography with a reverse phase column (TSKgel ODS-100 V, 150 × 4.6 mm ID, Tosoh) and a UV detector (GL-7450, GL science). The chromatographic conditions were as follows: flow rate, 1.0 mL min^−1^; detection wavelength, 273 nm; mobile phase, 50 : 50 (v/v) water/acetonitrile. A total organic carbon (TOC) analyser (TOC-V_E_, Shimadzu) was used to investigate the decrease in the TOC of the 2-CP solution during the photocatalytic reaction. For the radical scavenging test, various scavengers were added to the reaction system under the same experimental conditions as those used for the photocatalytic degradation experiment. A 30 mM ammonium oxalate (AO) solution, 30 mM *t*-butyl alcohol (TBA) solution, and 10 mM 4-hydroxy-2,2,6,6-tetramethylpiperidine (TEMPO) solution were used as scavengers for detecting the generation of h^+^, ·OH, and O_2_˙^−^, respectively.

#### Characterization

X-ray diffraction (XRD) patterns of the prepared samples were recorded on a powder X-ray diffractometer (Ultima IV, Rigaku) equipped with a Cu-Kα radiation source. Scanning electron microscopy (SEM) (S-4300, Hitachi) and transmission electron microscopy (TEM) (JEM-1011, JEOL) were performed to observe the morphologies of the samples. Energy dispersive X-ray spectrometry (EDX) mapping images were collected using an Ex-350 (Horiba). The surface properties of the samples were investigated by means of nitrogen adsorption–desorption experiments (BEL-SORP-miniII, Bel). The photoluminescence (PL) spectra of the photocatalysts were acquired using a fluorescence spectrophotometer (FP-8500, JASCO) at the excitation wavelength of 300 nm. In order to determine the chemical composition of the samples, X-ray photoelectron spectroscopy (XPS) was performed on an X-ray photoelectron spectrometer (PHI Quantera SXM, Ulvac-Phi), equipped with an Al-Kα radiation source. An UV-vis spectrophotometer (V-750, JASCO) was used to acquire the UV-visible diffuse reflectance spectra (UV-vis DRS) of samples. The infrared spectra in the range of 4000–500 cm^−1^ were acquired from a Fourier transform infrared (FTIR) spectrometer (Spectrum 100, PerkinElmer).

## Results and discussion

### Material characterization

**Fig. 1:**
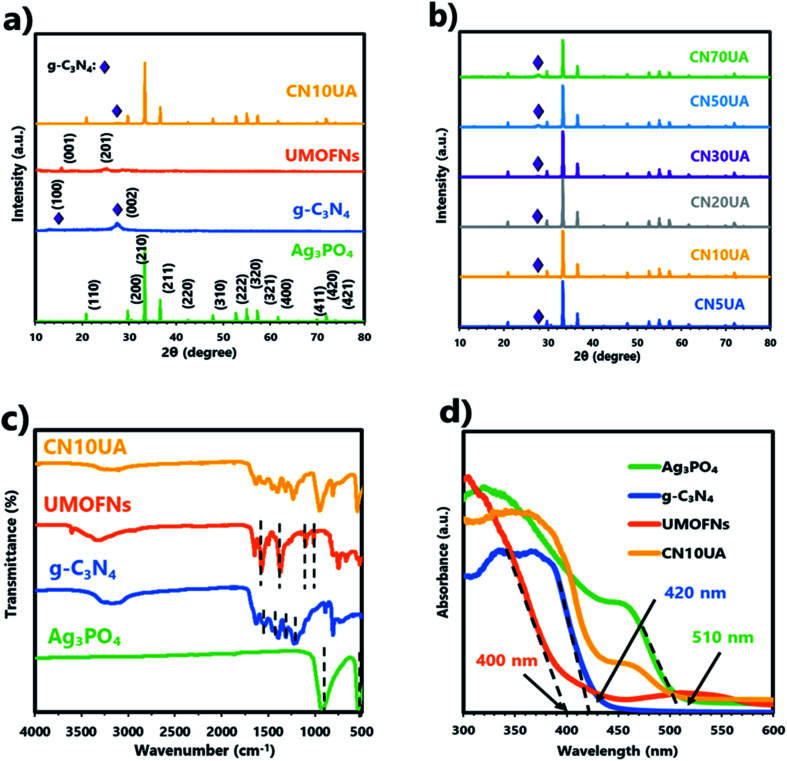
(a) XRD patterns of various photocatalytic materials. (b) XRD patterns of CNUA with different amounts of g-C_3_N_4_ (5, 10, 20, 30, 50, and 70%). (c) FTIR spectra and (d) UV-vis DRS of various photocatalytic materials.

In order to identify the crystal structure and composition of the as-synthesized materials, the XRD patterns of g-C_3_N_4_, UMOFNs, Ag_3_PO_4_, and CN10UA were analysed (Fig. 1a). For g-C_3_N_4_, the two characteristic diffraction peaks observed at 12.7 and 27.5° correspond to the (1 0 0) and (0 0 2) planes of the material, respectively. The former peak is attributed to the interlayer stacking of aromatic systems, while the latter one is attributed to in-plane structural packing.^44^ For UMOFNs, the same XRD pattern as that reported previously was obtained,^20^ confirming that UMOFNs were successfully synthesized. In the XRD pattern of Ag_3_PO_4_, the positions of all characteristic diffraction peaks are in good agreement with those of a body-centred cubic structure of Ag_3_PO_4_ (JCPDS no. 06-0505). In addition, the narrow and sharp peaks reveal that the prepared Ag_3_PO_4_ particles are highly crystalline without any other impurities. The intensity ratio between the signals of (2 2 2) and (2 0 0) planes is 0.96, suggesting that tetrahedral Ag_3_PO_4_ composed of {1 1 1} facets was successfully synthesized.^57^ In the XRD pattern of CN10UA, a weak peak derived from g-C_3_N_4_ and the characteristic peaks corresponding to Ag_3_PO_4_ could be observed, while no peaks attributable to UMOFNs were observed because of their lower concentration and relatively weaker crystallinity in comparison with those of Ag_3_PO_4_. This result is consistent with a previous report on UMOFN/Ag_3_PO_4_ photocatalyst.^56^ Moreover, no other characteristic diffraction peaks are observed, indicating that the CN10UA composite photocatalyst is a highly pure material without any impurities. The diffraction peaks of CNUA samples with different concentrations of g-C_3_N_4_ (5, 10, 20, 30, 50, and 70 wt%) are presented in Fig. 1b. As the weight ratio of g-C_3_N_4_ is gradually increased, the peak intensity derived from g-C_3_N_4_ becomes stronger in the XRD pattern of CNUA. These results indicate the successful preparation of CNUA systems with ternary heterojunctions.

The FTIR spectra of g-C_3_N_4_, UMOFNs, Ag_3_PO_4_, and CNUA with different concentrations of g-C_3_N_4_ (5, 10, 20, 30, 50, and 70 wt%) were also acquired to further determine the composition of the photocatalytic materials. As shown in Fig. 1c, the two characteristic peaks at 539 and 923 cm^−1^ in the FTIR spectrum of Ag_3_PO_4_ result from the bending vibration of P–O bond and asymmetric stretching vibration of PO_4_^3−^, respectively.^44^ The FTIR spectrum of UMOFNs shows two strong peaks at 1370 and 1647 cm^−1^ corresponding to the asymmetric and symmetric stretching vibrations of the carboxyl group in terephthalic acid, respectively.^56^ The two peaks at 1016 and 1102 cm^−1^ are attributed to the C–N stretching vibration of DMF. The appearance of these peaks indicates the presence of DMF solvent in the pores of UMOFNs because they could not be removed during the preparation of UMOFNs.^56^ The peak at ∼600–800 cm^−1^ is attributed to the vibration of the metal–oxygen bond in UMOFNs.^56^ In the case of g-C_3_N_4_, the characteristic peaks located at 1227, 1312, 1454, 1536, and 1628 cm^−1^ could be attributed to the C–N stretching vibration.^44^ Further, the peak at 807 cm^−1^ and the broad peak at ∼3200 cm^−1^ are attributed to the breathing of triazine units and N–H stretching vibration, respectively.^44^ In the CN10AU composite photocatalyst, prominent peaks corresponding to g-C_3_N_4_ and Ag_3_PO_4_ appeared, while no peaks attributed to UMOFN are observed due to its lower concentration in comparison with that of Ag_3_PO_4_. Furthermore, these peaks are slightly shifted to higher wave numbers, suggesting the presence of weak interactions between g-C_3_N_4_, UMOFNs, and Ag_3_PO_4_ (Fig. S1†).^20^ These results are in good agreement with those of the XRD analysis.

**Fig. 2:**
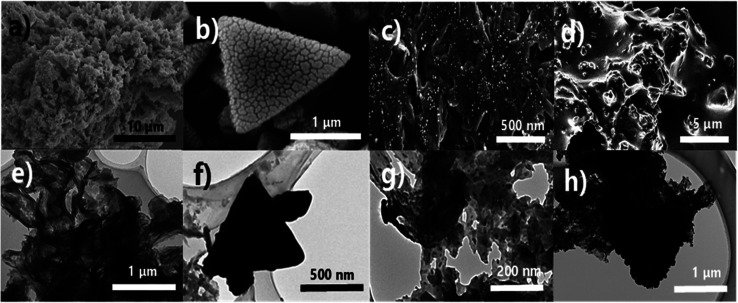
SEM (a–d) and TEM (e–h) images of (a and e) g-C_3_N_4_ (b and f) Ag_3_PO_4_, (c and g) UMOFNs, and (d and h) CN10UA.

The surface morphology of the photocatalytic materials was observed by SEM and TEM. Fig. 2a and e show that g-C_3_N_4_ has an irregularly stacked structure. Further, Fig. 2b and f show that Ag_3_PO_4_ particles have a tetrahedral morphology with 1 μm side length and exposed {1 1 1} facets, which have higher surface energy than other facets.^57^Fig. 2c and g show that UMOFNs are ultra-thin nanosheets with slightly rolled edges. Finally, the SEM images of CN10UA in Fig. 2d and h reveal that Ag_3_PO_4_ tetrahedrons are incorporated into g-C_3_N_4_ without undergoing any morphological changes. However, UMOFNs cannot be identified due to their ultra-thin two-dimensional nanosheet structure. In addition, EDX mapping analysis was performed to further confirm the distribution of elements in CN10UA, (Fig. S2†). As shown in Fig. S2,† C, N, O, Co, Ni, Ag and P elements exist uniformly in CN10UA, indicating that the hetero-structure of the ternary photocatalyst is composed of UMOFNs, g-C_3_N_4_ and Ag_3_PO_4_ without other impurities.

The porosity and specific surface area of photocatalytic materials were determined by the analysis of nitrogen adsorption–desorption isotherms. As shown in Fig. S3,† CN10UA demonstrates a type IV isotherm according to the IUPAC classification, confirming that it has both mesopores and micropores. In addition, the Brunauer–Emmett–Teller (BET) surface areas of materials used in this study are summarized in Table 1. The BET surface area of Ag_3_PO_4_ is 0.78 m^2^ g^−1^. With an increase in the g-C_3_N_4_ content, the BET surface area of CNUA increases from 3.66 m^2^ g^−1^ (CN5UA) to 9.65 m^2^ g^−1^ (CN10UA), 10.11 m^2^ g^−1^ (CN20UA), 10.13 m^2^ g^−1^ (CN30UA), 18.26 m^2^ g^−1^ (CN50UA), and 26.88 m^2^ g^−1^ (CN70UA). It is well known that a photocatalyst with a higher BET surface area facilitates effective adsorption of organic pollutants onto its surface and provides more photocatalytic active sites. Therefore, it is reasonable to assume that the composite catalyst would improve the efficiency of the photocatalytic reaction because the surface area of the material increased with the introduction of g-C_3_N_4_ into Ag_3_PO_4_.

**Table 1:** BET surface area, pore volume, and pore diameter of as-prepared photocatalysts

Samples	BET surface area (m^2^ g^−1^)	Pore volume (cm^3^ g^−1^)	Pore diameter (nm)
Ag_3_PO_4_	0.78	0.0015	7.82
UMOFNs	6.32	0.0196	12.63
g-C_3_N_4_	89.24	0.6521	29.22
UA	1.60	0.0045	11.36
CAN	5.06	0.0235	18.65
CN5UA	3.66	0.0143	15.67
CN10UA	9.65	0.0788	32.65
CN20UA	10.11	0.0756	29.91
CN30UA	10.13	0.0861	33.45
CN50UA	18.26	0.1481	32.45
CN70UA	26.88	0.2122	31.55

The optical properties and band structures of samples were studied by UV-vis diffuse reflectance spectroscopy. As shown in Fig. 1d, the adsorption edge of g-C_3_N_4_, UMOFNs, and Ag_3_PO_4_ were determined to be 420, 380, and 510 nm, respectively. Based on the Kubelka–Munk formula, the band gap energies (*E*_g_) of g-C_3_N_4_, UMOFNs, and Ag_3_PO_4_ were calculated to be 2.89, 3.01, and 2.43 eV, respectively (Fig. S4a†). In addition, valence band (VB) XPS analysis was performed to estimate the position of the valence band edge (*E*_VB_). For g-C_3_N_4_, UMOFNs, and Ag_3_PO_4_, the positions of the *E*_VB_ were determined to be 1.43, 1.62, and 2.61 eV, respectively (Fig. S4b†). Therefore, their conduction band (*E*_CB_) positions were calculated to be −1.46, −1.39, and +0.18 eV, respectively, according to the empirical equation.

**Fig. 3:**
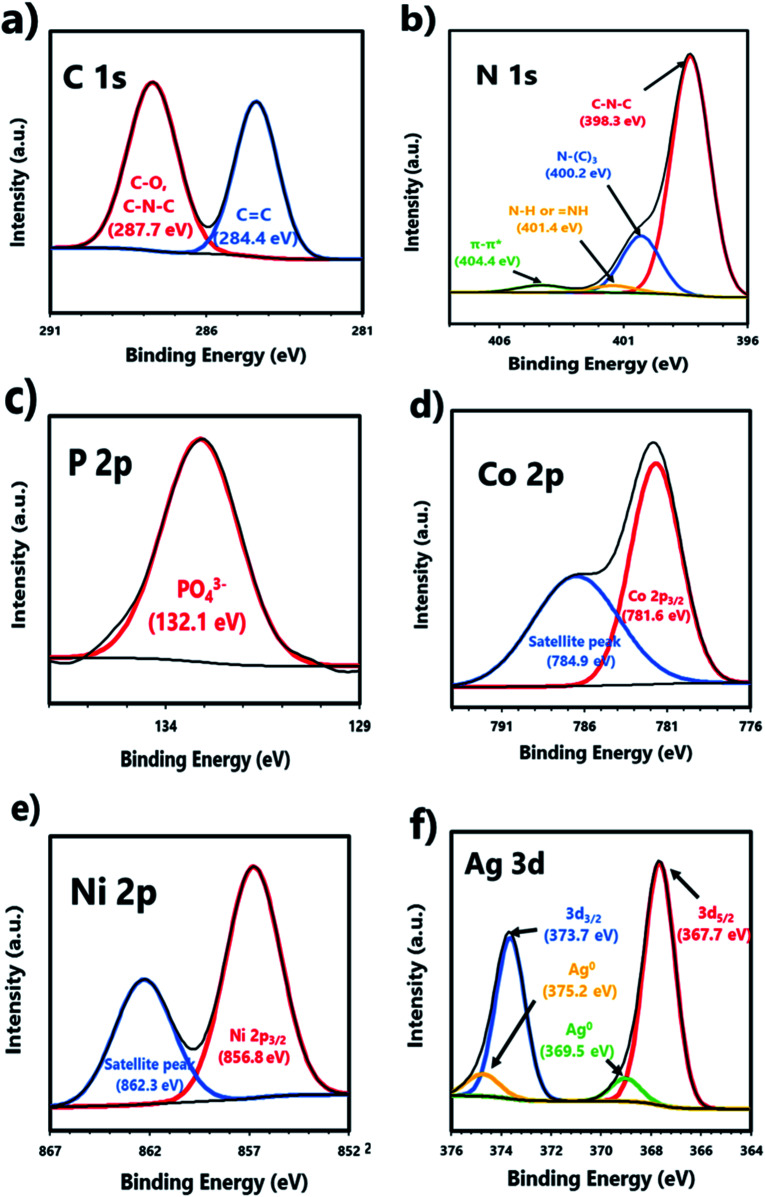
XPS spectra of CN10UA: (a) C 1s, (b) N 1s, (c) P 2p, (d) Co 2p, (e) Ni 2p, and (f) Ag 3d spectra.

XPS analysis was performed to verify the chemical composition and environments of CN10UA (Fig. 3 and S5†). In Fig. S5a,† the XPS survey spectra indicates the existence of C, N, O, P, Co, Ni, and Ag in the ternary system, thus suggesting the coexistence of g-C_3_N_4_, UMOFNs, and Ag_3_PO_4_ in the ternary composite photocatalyst. Furthermore, other characteristic peaks are not observed, indicating that the ternary CN10UA composite is a high-purity photocatalyst without any impurities. As shown in Fig. 3a, the C 1s narrow-scan XPS spectrum has two strong peaks at 284.4 and 287.7 eV corresponding to sp^2^-hybridized carbons (C=C) in aromatic hydrocarbons and sp^2^-hybridized nitrogen atoms (C=N) in the triazine ring of g-C_3_N_4_ and/or carbonate species (C–O) in UMOFNs, respectively.^44,56^ In the N 1s narrow-scan XPS spectrum in Fig. 3b, four peaks located at 398.3, 400.2, 401.4, and 404.4 eV are attributed to sp^2^-hybridized N (C=N–C), sp^3^-hybridized nitrogen (N–(C_3_)), amino functional groups (N–H and =NH) in g-C_3_N_4_, and the charge effects, respectively.^44^ The O 1s peaks are located at 530.8 and 532.5 eV in Fig. S5b.† The former peak is assigned to the lattice oxygen in Ag_3_PO_4_ and/or metal–oxygen bonds (Ni–O and Co–O) in UMOFNs.^44,56^ Further, the latter peak is assigned to the hydroxyl groups or water molecules adsorbed on the surface of CN10UA.^44^Fig. 3c presents the P 2p peak at 132.1 eV, which is derived from PO_4_^3−^ in Ag_3_PO_4_.^44^ Further, the two peaks at 781.6 and 784.9 eV in the Co 2p narrow-scan XPS spectrum (Fig. 3d), which could be attributed to Co 2p_3/2_ and its satellite peak, respectively, indicate the presence of Co^2+^ in UMOFNs.^56^ In Fig. 3e, the two peaks in the Ni 2p narrow-scan XPS spectrum are located at 856.8 and 862.3 eV, which correspond to Ni 2p_3/2_ and its satellite peak, respectively, suggesting the existence of Ni^2+^ in UMOFNs.^56^ As shown in Fig. 3f, there are two strong peaks and two weak peaks in the Ag narrow-scan XPS spectrum. The former peaks at 367.7 and 373.7 eV are attributed to Ag_5/2_ and Ag_3/2_ levels of Ag^+^ ions in Ag_3_PO_4_, respectively. Meanwhile, the latter peaks at 369.5 and 375.2 eV are attributed to the Ag^0^ nanoparticles; these peaks indicate the formation of Ag^0^ due to the migration of electrons from g-C_3_N_4_ to Ag^+^ ions during the synthesis of the composite photocatalysts.^44,56^ The trace amounts of Ag nanoparticles in CNUA serve both as an electron mediator and as a photosensitizer (owing to the surface plasm on resonance (SPR)) in the photocatalytic reaction.^58^

### Photocatalytic activity of the ternary photocatalysts

**Fig. 4:**
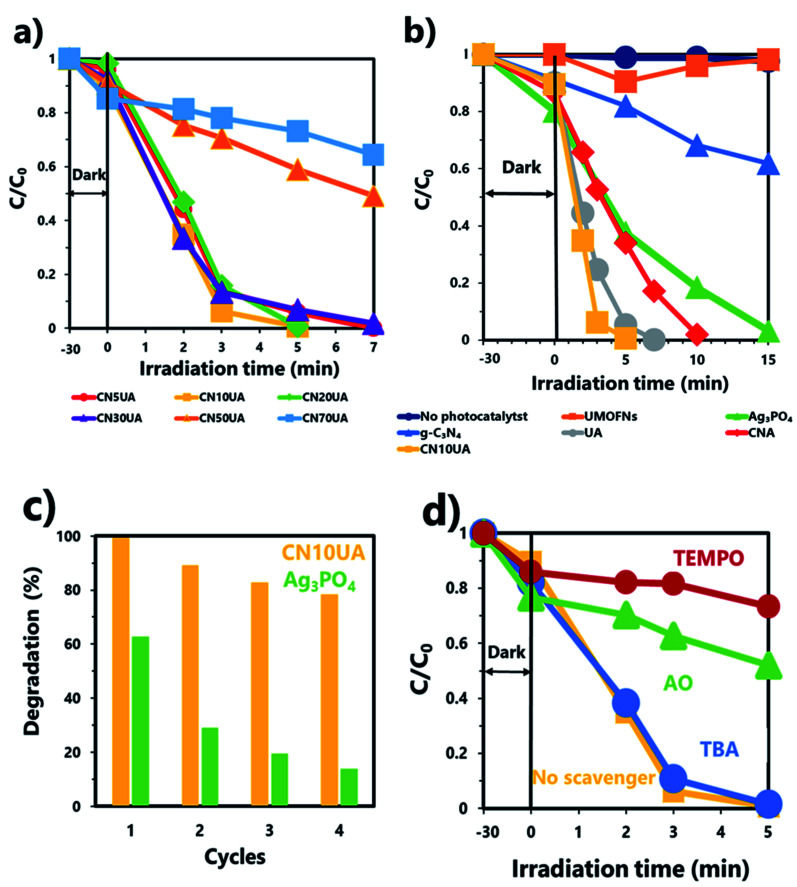
Photocatalytic activity of (a) CNUA samples with different amounts of g-C_3_N_4_ (5, 10, 20, 30, 50, and 70%) and (b) different photocatalysts (single component, binary, and ternary) under visible-light irradiation. (c) Results of the recycling of Ag_3_PO_4_ and CN10UA in the photodegradation of 2-CP. (d) Effect of various radical scavengers on the photocatalytic activity of CN10UA.

The photocatalytic performance of the as-synthesized samples was determined by evaluating the efficiency of photodegradation of 2-CP under their catalysis. Fig. 4a shows the photocatalytic activity of CNUA samples with different amounts of g-C_3_N_4_ (5, 10, 20, 30, 50, and 70%). Among the ternary photocatalysts, CN10UA exhibited the best photocatalytic activity. In addition, the apparent first-order rate constants (*k*_app_) for a series of CNUA samples were calculated using the pseudo-first-order kinetics equation. The value of *k*_app_ follows the order of CN70UA (0.06 min^−1^) < CN50UA (0.10 min^−1^) < CN30UA (0.56 min^−1^) < CN5UA (0.57 min^−1^) < CN20UA (0.84 min^−1^) < CN10UA (0.93 min^−1^), as shown in Fig. S6a.† Apparently, CN10UA has the highest *k*_app_ value among the prepared CNUA samples. Thus, the optimal amount of g-C_3_N_4_ required to form the ternary system was determined to be 10 wt%. Fig. 4b shows that 2-CP did not undergo any degradation in the absence of photocatalysts, indicating that its self-photolysis reaction during the photocatalytic process is negligible and could be neglected. In the presence of pure Ag_3_PO_4_, the photocatalytic efficiency of 2-CP reached 97.1% within 15 min. When Ag_3_PO_4_ is combined with UMOFNs or g-C_3_N_4_, the photocatalytic activity of the binary photocatalyst, UA or CNA, is improved as compared to that of pure Ag_3_PO_4_. Remarkably, after the formation of the ternary system consisting of g-C_3_N_4_, UMOFNs, and Ag_3_PO_4_, the photocatalytic activity increased significantly. The ternary photocatalyst showed higher photocatalytic activity than those of the binary or single-component photocatalysts, and completely degraded 2-CP in only 5 min. The enhanced photocatalytic activity of ternary photocatalysts results from more efficient and fast charge separation through dual *Z*-scheme mechanism, as discussed in the following section. The TOC removal rate was also analysed to investigate the degree of mineralization of 2-CP over Ag_3_PO_4_ and CN10UA. As shown in Fig. S7,† TOC removal efficiencies of 22.1 and 98.7% in 120 min were achieved using Ag_3_PO_4_ and CN10UA as photocatalysts, respectively, suggesting that the construction of the ternary system also led to enhanced photocatalytic mineralization activity. This result is in good agreement with the results of the photocatalytic degradation.

The reusability of the photocatalyst is a very important factor for its practical application, and recycling experiments were carried out using pure Ag_3_PO_4_ and CN10UA to evaluate their reusability. As shown in Fig. 4c, pure Ag_3_PO_4_ showed poor photocatalytic stability during the 2-CP photodegradation. However, the photocatalytic efficiency of CN10UA remained as high as 78.1% even after four reaction cycles, indicating that the construction of the ternary system also led to improved stability of the catalyst under visible light. In addition, the recycled photocatalyst was characterized by XRD, SEM and DRS to investigate the stability and possible reaction mechanism. The XRD pattern of the used CN10UA photocatalyst in Fig. S8a† shows the appearance of a relatively weak diffraction peak at 38.1° corresponding to the (1 1 1) plane of Ag^0^ (JCPDS no. 65-8425). This result suggests that Ag^+^ ions in the Ag_3_PO_4_ are reduced to produce additional Ag^0^ nanoparticles on CNUA during photocatalysis. That is, Ag^+^ in CNUA is reduced to Ag^0^ not only during the synthetic process but also during the photocatalytic degradation of 2-CP. On the other hand, a stronger peak at 38.1° compared to that of used CN10UA and two weak peaks at 44.3 and 64.3° can be observed in the XRD patterns of the used Ag_3_PO_4_, corresponding to (1 1 1), (2 0 0), and (2 2 0) planes of Ag^0^, respectively. These differences indicate that CN10UA has higher photo-corrosion resistance as compared to pure Ag_3_PO_4_ due to the consumption of electrons from the conduction band (CB) of Ag_3_PO_4_. The SEM images of the used photocatalyst in Fig. S9† show that the surface morphology of CN10UA remains almost the same as that of a fresh one after four reaction cycles. The DRS of the used photocatalyst in Fig. S8b† shows that the intensity of the absorption band in the visible range is significantly increased after visible-light irradiation due to the formation of additional Ag^0^ species during the photocatalytic reaction. In general, it is known that an excessive amount of Ag nanoparticles interferes with light adsorption, contact of the catalyst with organic pollutants, and charge migration on Ag_3_PO_4_, thereby decreasing the photocatalytic performance.^56^ Therefore, the slight deterioration of the photocatalytic activity during the cycling experiments can be attributed to the deposition of additional Ag^0^ nanoparticles on the photocatalyst during the photocatalytic process.

### Possible photocatalytic mechanism

To investigate the main reactive species involved in the photocatalytic degradation of 2-CP over CN10UA, radical scavenging tests were conducted under the same experimental conditions as the photocatalytic degradation experiment. AO, TBA, and TEMPO were added to the reaction system for detecting the generation of h^+^, ·OH, and O_2_˙^−^, respectively. Fig. 4d shows that the addition of TBA had no effect on the degradation reaction, while the addition of AO and TEMPO led to the suppression of the photocatalytic degradation. In addition, the values of *k*_app_ were calculated in Fig. S6b,† and their order was as follows: TEMPO (0.03 min^−1^) < AO (0.09 min^−1^) < TBA (0.75 min^−1^) < No scavenger (0.93 min^−1^). These results indicate that h^+^ and O_2_˙^−^ play an important role as the main reactive species in the photocatalytic degradation of 2-CP. In particular, O_2_˙^−^ played a more significant role in the degradation of 2-CP.

**Fig. 5:**
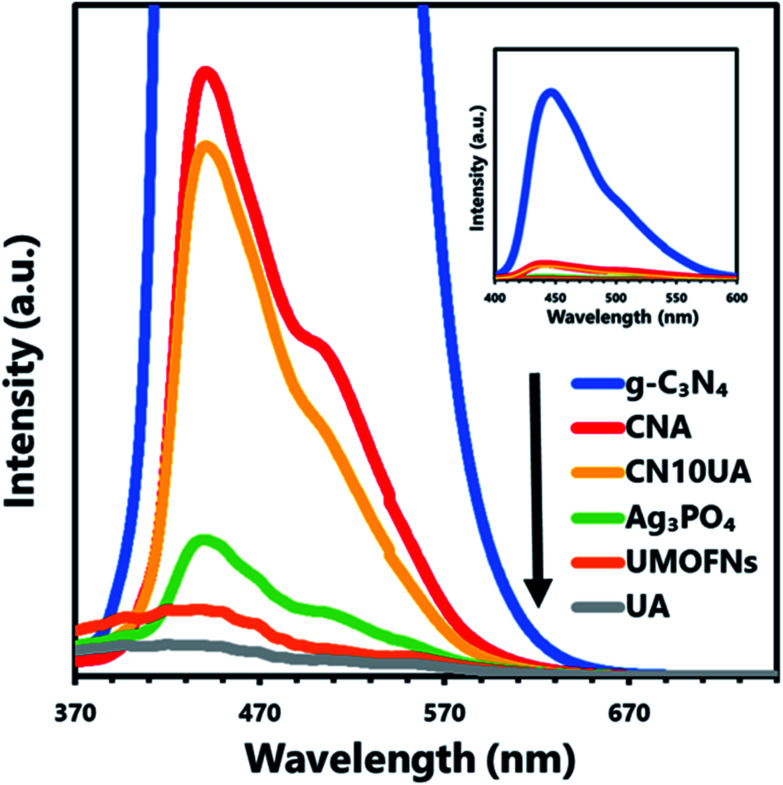
Photoluminescence spectra of various photocatalytic materials.

Furthermore, PL analysis was performed to further investigate the charge transfer pathway in the ternary composite. In Fig. 5, the PL spectrum of the binary CNA photocatalyst shows higher intensity than that of pure Ag_3_PO_4_, indicating the fast recombination rate of photogenerated electrons in the CB of Ag_3_PO_4_ and holes in the VB of g-C_3_N_4_ through the Ag bridge; that is, g-C_3_N_4_/Ag/Ag_3_PO_4_ pathways were constructed in CNA. A similar phenomenon was observed in a previous work.^44^ The PL intensity of ternary CN10UA photocatalyst is lower than that of binary CNA, because of the efficient transport of photogenerated electron–hole pairs *via* Ag electron separation centres in Ag_3_PO_4_/Ag/UMOFNs pathways.^49,56^ Taking these results into account, we infer that the photogenerated electrons on the ternary CNUA photocatalysts are transferred through dual *Z*-scheme pathways during the photocatalytic reaction, leading to the efficient charge separation of photoinduced electron–holes on the ternary CNUA composite photocatalysts.

**Fig. 6:**
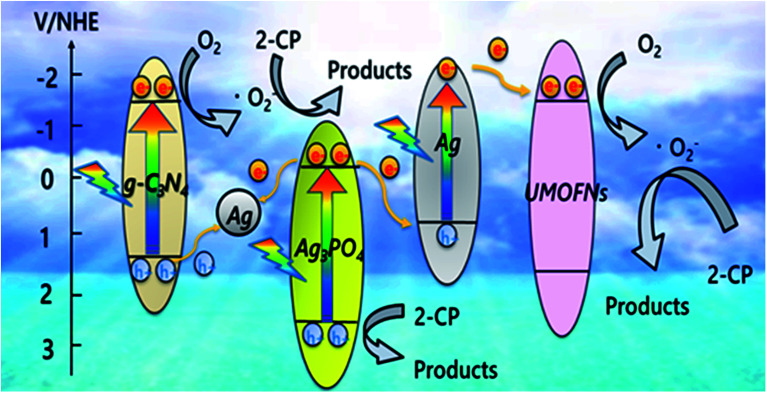
Possible reaction mechanism of the photodegradation of 2-CP over CN10UA under visible-light irradiation.

Based on the results of PL, DRS, XPS, and radical trapping studies on the ternary photocatalyst, a possible reaction mechanism of 2-CP photodegradation *via* the dual *Z*-scheme pathway over CN10UA under-visible light irradiation is proposed, as illustrated in Fig. 6. First, the trace amounts of Ag nanoparticles formed during the photocatalyst preparation are deposited on the composite due to the migration of electrons from g-C_3_N_4_ to Ag_3_PO_4_, as evidenced from XPS analysis. These Ag nanoparticles play an important role as dual *Z*-scheme channels in the g-C_3_N_4_/Ag/Ag_3_PO_4_ and Ag_3_PO_4_/Ag/UMOFNs pathways. Moreover, the Ag nanoparticles can be activated through the SPR effect under visible-light irradiation. In the g-C_3_N_4_/Ag/Ag_3_PO_4_ system, g-C_3_N_4_ and Ag_3_PO_4_ can be activated by visible light to generate electron–hole pairs. The photogenerated electrons in the CB of Ag_3_PO_4_ are easily transferred to the Ag metal, and then they recombine with the photogenerated holes from the VB of g-C_3_N_4_*via* the Ag bridge. As a result, the electrons left on the g-C_3_N_4_ reduce O_2_ to O_2_˙^−^, which oxidizes 2-CP. Meanwhile, the holes on Ag_3_PO_4_ directly degrade 2-CP owing to the great ability of PO_4_^3−^ to attract the 2-CP electrons and the high positive potential of the VB that mainly consists of Ag 4d and O 2p orbitals.^57^ In the other system of Ag_3_PO_4_/Ag/UMOFNs, Ag nanoparticles absorb the visible light as a photosensitizer, and aids the formation of electron–hole pairs by the SPR effect. The SPR-induced electrons migrate to the CB of UMOFNs to reduce O_2_ to O_2_˙^−^. In contrast, the SPR-induced holes recombine with the photogenerated electrons from Ag_3_PO_4_ due to the lower Fermi level of Ag nanoparticles (*E*_f_ = +0.80 V *vs.* NHE).^43^ Therefore, the dual *Z*-scheme could facilitate more positive and negative potentials of VB and CB, respectively, which are favourable for the oxidation of 2-CP and the reduction of O_2_, respectively. Moreover, the electrons in the CB of Ag_3_PO_4_ flow into the Ag nanoparticles through the two *Z*-scheme pathways, instead of remaining in the CB of Ag_3_PO_4_, which could prevent the self-reduction of Ag_3_PO_4_ and the production of H_2_O_2_ from the reduction of O_2_ also occurred on the CB of Ag_3_PO_4_ through a two-electron process (*E*° (O_2_/H_2_O_2_) = +0.68 V *vs.* NHE).^59,60^ Consequently, the dual *Z*-scheme-type ternary CN10UA photocatalyst facilitates fast charge separation, leading to its high stability and enhanced photocatalytic activity in 2-CP photodegradation.

## Conclusions

We successfully synthesized ternary CNUA photocatalysts *via* ultrasound irradiation in a THF solution. The photocatalytic activity of the as-prepared materials was evaluated by employing these in the photodegradation of 2-CP under visible light irradiation. The ternary CN10UA photocatalyst shows better photocatalytic activity in comparison with binary or single-component photocatalysts; it facilitated almost complete degradation of 2-CP in only 5 min. Moreover, CN10UA also demonstrated high stability and effective charge separation. The observed enhancement in the photocatalytic activity of CN10UA results from fast charge transport through dual *Z*-scheme channels, *via* the g-C_3_N_4_/Ag/Ag_3_PO_4_ and Ag_3_PO_4_/Ag/UMOFNs pathways. In this system, the Ag bridges served both as an electron mediator and a photosensitizer *via* the SPR effect, and they remarkably promoted the rate of charge transport. In addition, PL analysis confirmed the transition of the photogenerated electrons on the ternary CNUA photocatalysts through dual *Z*-scheme pathways during the photocatalytic reaction. The findings of this study are expected to contribute to a better understanding of the construction and mechanism of dual *Z*-scheme type photocatalysts.

## Conflicts of interest

There are no conflicts to declare.

## Supplementary Material

RA-009-C9RA08292A-s001
