# Supplementary material for: Ternary dual Z-scheme graphitic carbon nitride/ultrathin metal–organic framework nanosheet/Ag3PO4 photocatalysts for boosted photocatalytic performance under visible light
Source: RSC Adv. 2019 Dec 2;9(68):39843–53. doi: 10.1039/c9ra08292a (PMC9076175; doi:10.1039/c9ra08292a)
Supplement: RA-009-C9RA08292A-s001 [file RA-009-C9RA08292A-s001.pdf]

Electronic Supplementary Material (ESI) for RSC Advances.
This journal is © The Royal Society of Chemistry 2019

Supporting Information

Ternary dual Z-scheme graphitic carbon nitride/ultrathin metal-organic framework nanosheet/Ag_3PO_4 photocatalysts for boosted photocatalytic performance under visible light

Tomoharu Kusutaki,[a] Hideyuki Katsumata,[*,a] Ikki Tateishi,[b] Mai Furukawa,[a] and Satoshi Kaneco[a,b]

[a] Department of Chemistry for Materials, Graduate School of Engineering, Mie University, Tsu, Mie 514-8507, Japan
[b] Mie Global Environment Center for Education & Research, Mie University, Tsu, Mie 514-8507, Japan

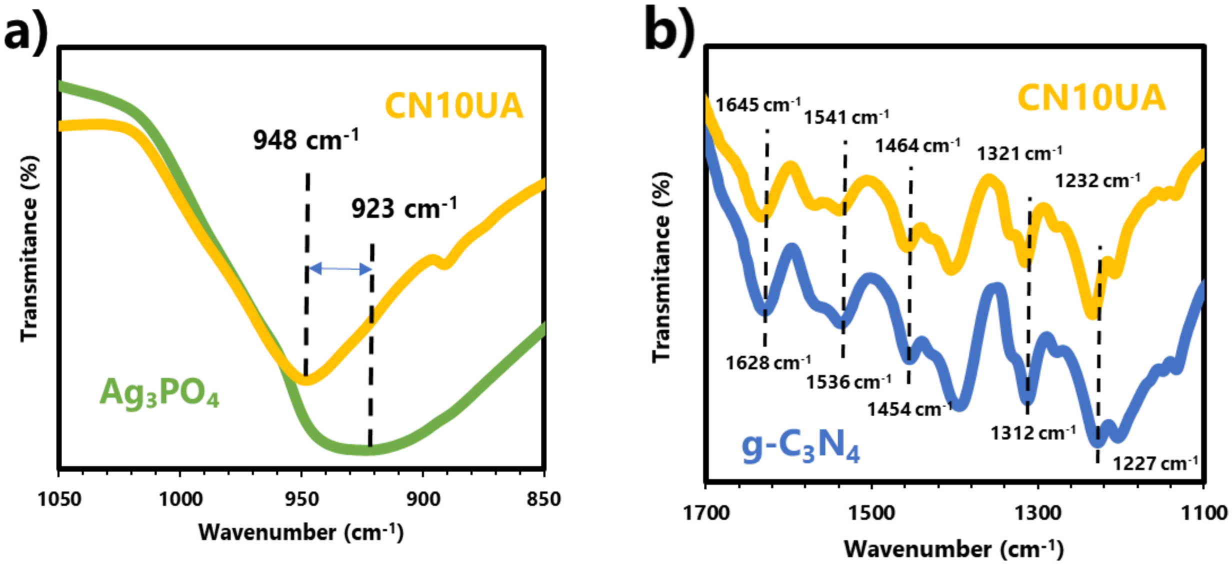

Figure S1. FTIR spectra of (a) Ag_3PO_4 and (b) g-C_3N_4 comparison with CN10UA spectra.

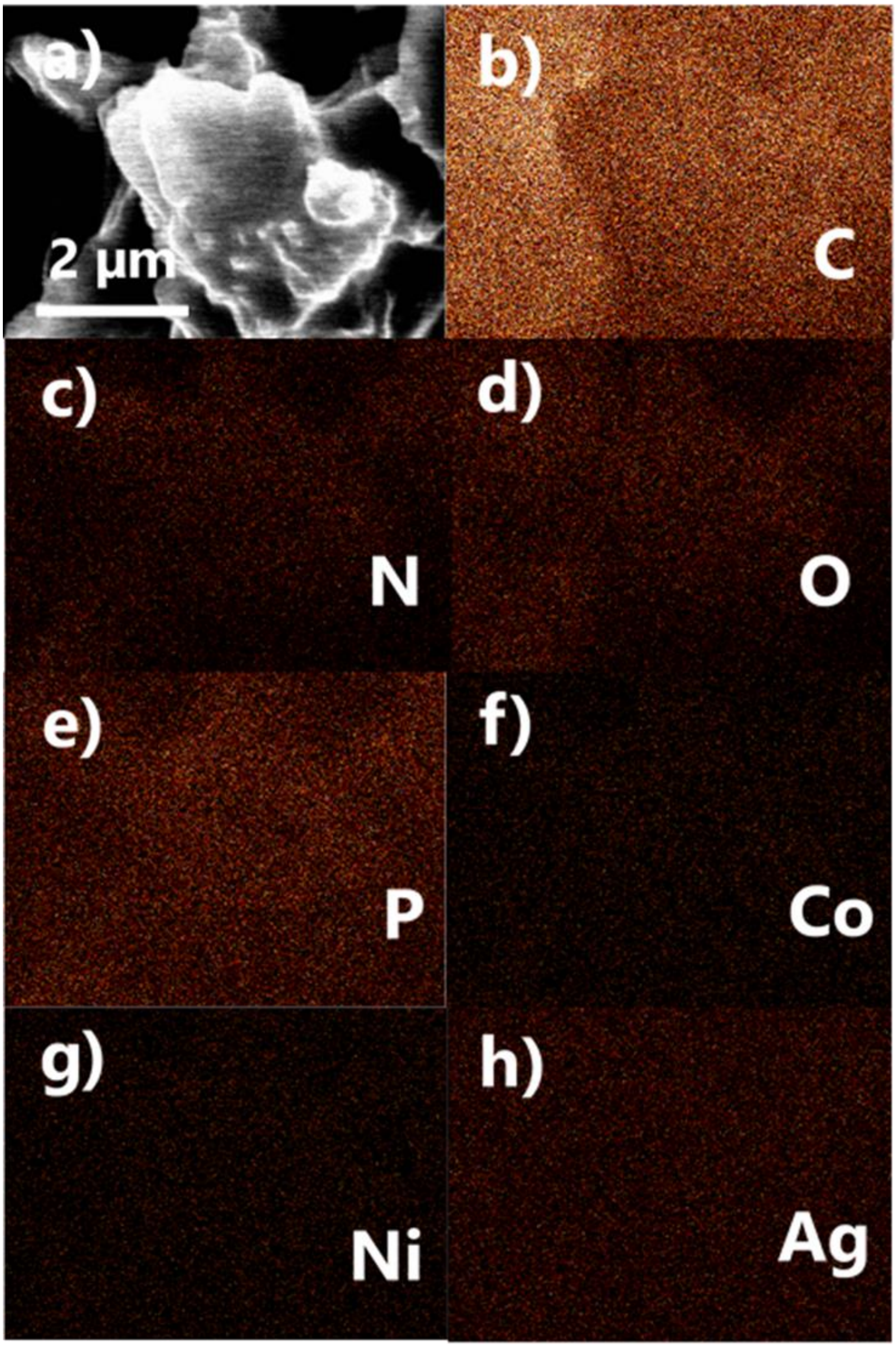

Figure S2. (a) SEM and (b-f) EDX mapping images of CN10UA.

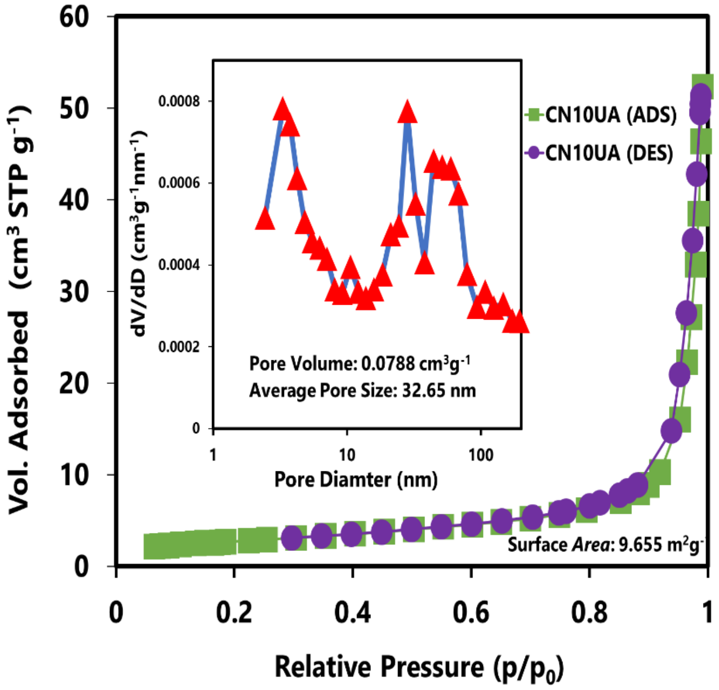

Figure S3. N_2 adsorption-desorption isotherm and pore size distribution curve for CN10UA.

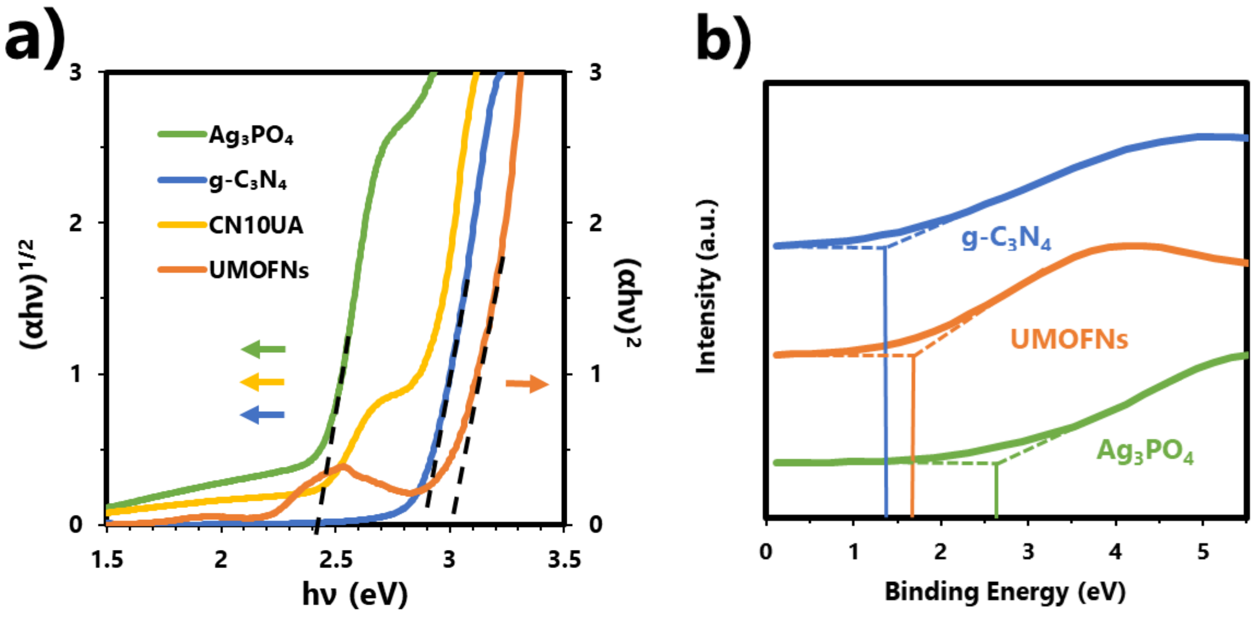

Figure S4. (a) Tauc plots, that is, $(\alpha h\nu)^{1/2}$ vs. $h\nu$ plots for Ag_3PO_4, g-C_3N_4, CN10UA and UMOFNs. (b) VBXPS spectra of g-C_3N_4, UMOFNs, and Ag_3PO_4.

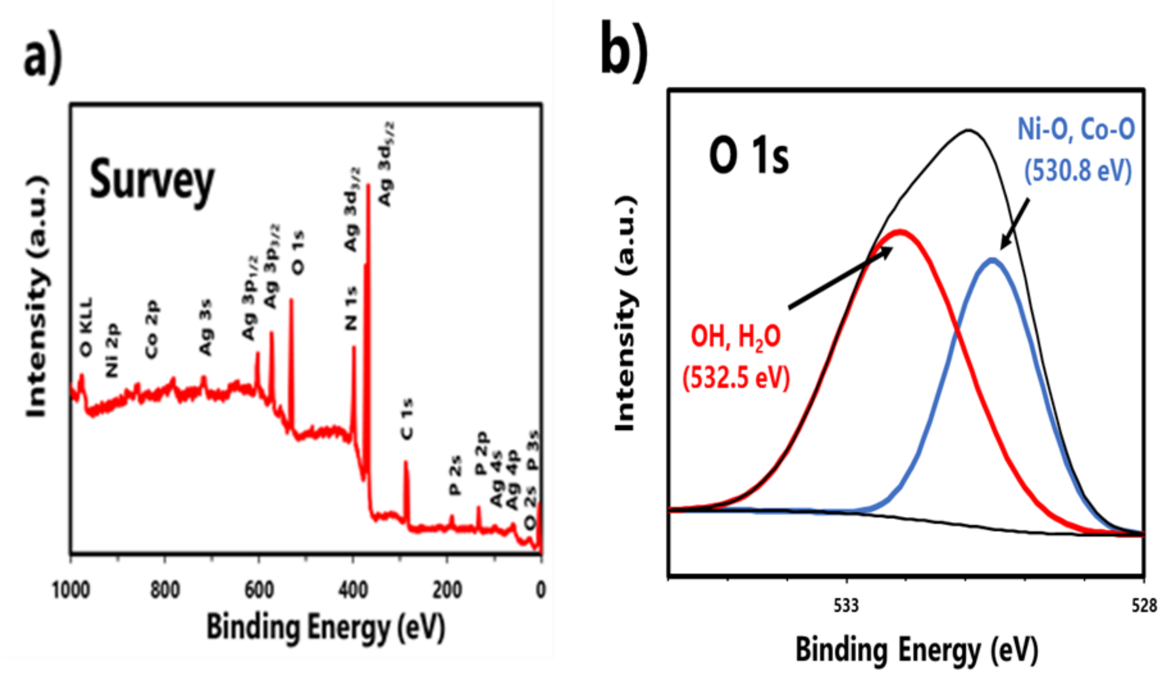

Figure S5. XPS spectra of CN10UA: (a) Survey spectrum and (b) O 1s.

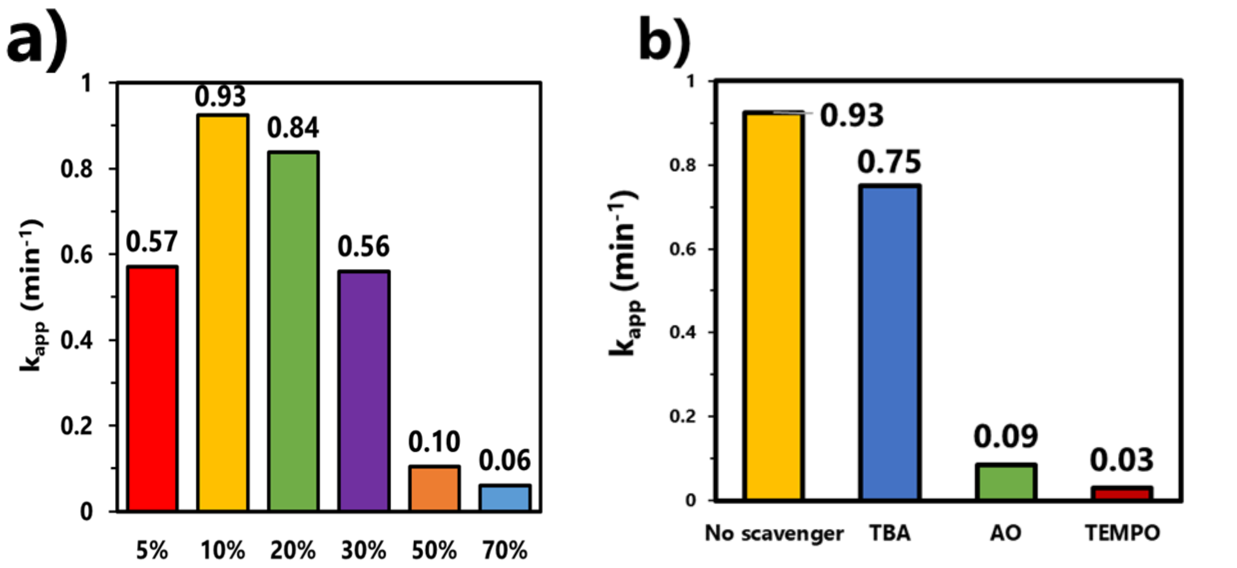

Figure S6. Pseudo-first order kinetic constants of (a) CNUA with different amounts of g-C_3N_4 (5, 10, 20, 30, 50, and 70%) and (b) CN10UA in the presence of various radical scavengers

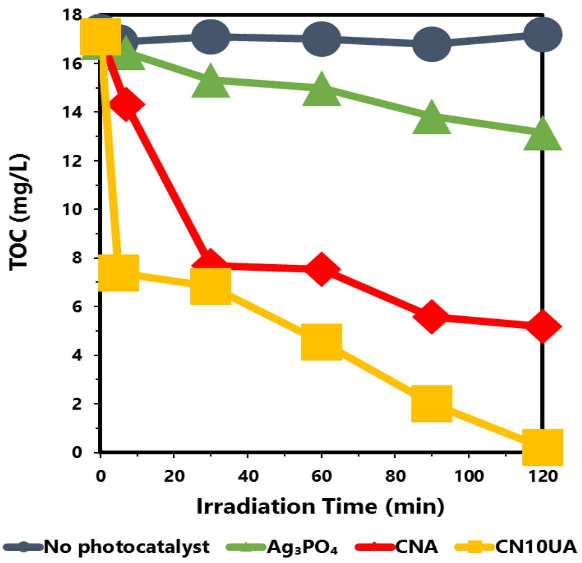

Figure S7. TOC removal of 2-CP over pure Ag_3PO_4, CNA and CN10UA.

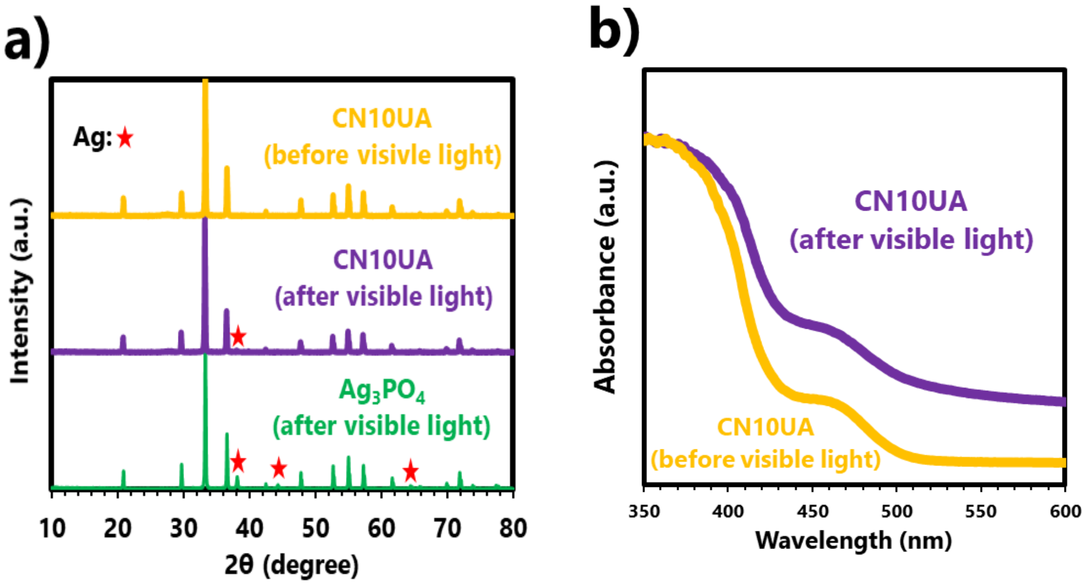

Figure S8. (a) XRD patterns (b) DRS of photocatalysts before and after visible light irradiation.

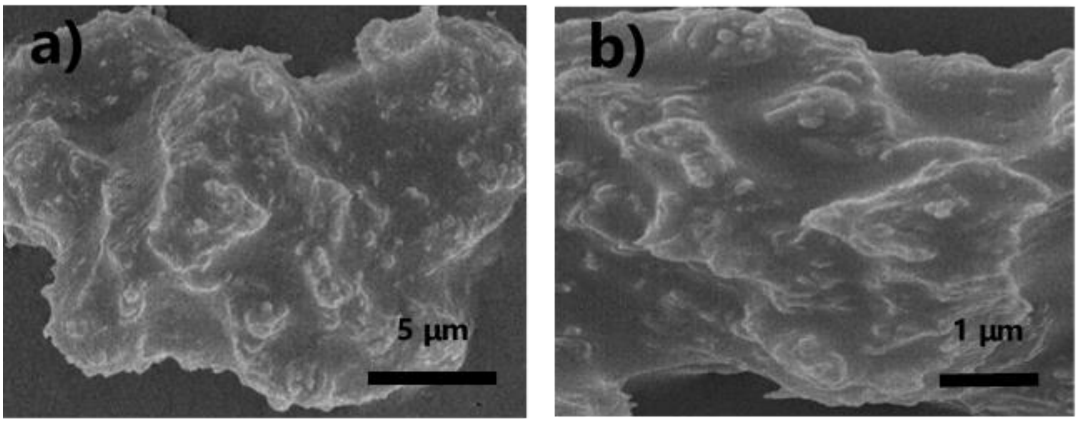

Figure S9. (a, b) SEM images of the used CN10UA.
